# Double-frequency grating shearing interferometer with built-in phase-shifting function for robust multi-level phase retrieval

**DOI:** 10.1038/s41598-022-13578-3

**Published:** 2022-06-08

**Authors:** Yeh-Wei Yu, Tsung-Yi Hou, Tsung-Hsun Yang, Ching-Cherng Sun

**Affiliations:** 1 Department of Optics and Photonics, National Central University, Chung-Li, 32001 Taiwan; 2 Department of Electrophysics, National Yang Ming Chiao Tung University, Hsin-Chu, 30010 Taiwan

**Keywords:** Optics and photonics, Physics

## Abstract

In this paper, we propose and demonstrate a novel interferometer and signal process to retrieve two-dimensional signals with multilevel phases. The interferometer is based on a shearing interferometry with double-frequency grating, and phase-shifting interferometry is derived as a built-in function of the lateral displacement of the grating. The interferometer not only retrieves the multilevel phase signals but also eliminates slow-varying phase errors wherever they occur. Owing to the common path algorithm, the new interferometer is more robust in dynamic circumstances for optical testing and data processing. We propose a pre-integral signal process for two-dimensional (2D) data processing to prevent post-phase-integral due to shearing interferometry. The simulation and experiment showed that the proposed interferometer with a pre-integral process has various advantages in signal processing for multilevel phase retrieval, and will be useful for higher data rates in optical data storage and free-space communication.

## Introduction

Multi-level phase encoding/modulation in a 2D phase array is very important in parallel optical information processing applied to data storage^1–9^, optical communication^10–24^, and adaptive optics^25–31^. The precise encoding of the phase in a dynamic environment and the decoding of the phase in a noisy environment are two major issues. System noise is a key barrier in the decoding part. One effective way to decode a 2D array with a multilevel phase signal is to apply phase-shifting interferometry (PSI)^32^. There have been various approaches to PSI, including 2-step, 3-step, 4-step, and 5-step algorithms^33–38^. However, a reference wave to perform phase shifting is necessary for the PSI. The reference wave requires high phase stability, a highly accurate phase-shifting, and a well-controlled wavefront. A fluctuation in the above three items can induce a phase error in the retrieved phase signal^32,39^. By contrast, shearing interferometry (SI) is an effective way to obtain relative phase information without additional reference waves because SI is a method to make an interferogram with self-interference^40–42^. There have been several effective methods to perform SI^43–52^. One of them is to use a double-frequency grating (DFG) to shear the diffracted waves when the wavefront passes through a designed grating, and is referred to as double-frequency grating shearing interferometry (DFGSI)^53,54^. The shearing amount and shearing orientation in the DFGSI can be controlled by an appropriate design of the grating. However, one of the major shortcomings of the conventional SI is that the relative-phase retrieval process assumes a spatially uniform distribution of the input amplitude^55,56^. When applied to the optical information process, even a smooth amplitude variation causes major noise in the retrieved relative phase. The second key limitation of an SI is that the relative phase before the integral along the shearing direction is not the true one. Thus, if phase errors occur at a certain location, the phase error will accrue at all pixels along the shearing direction, along with the post integral. By contrast, a pre-integral approach aimed at compensating for these two shortcomings has been proposed and demonstrated in a holographic data storage system^57^. This paper proposes and demonstrates a novel and powerful DFGSI with several unique advantages: (1) the self-reference shearing interferometer is robust in dynamic circumstances; (2) the shearing interferometer eliminates slow-varying phase errors; (3) the utilization of a built-in phase-shifting function combined can retrieve multi-level phase signals; (4) the pre-integral signal processing makes the DFGSI free from error accumulation and is more robust to retrieve multi-level phase signals.

## Principle

**Figure 1:**
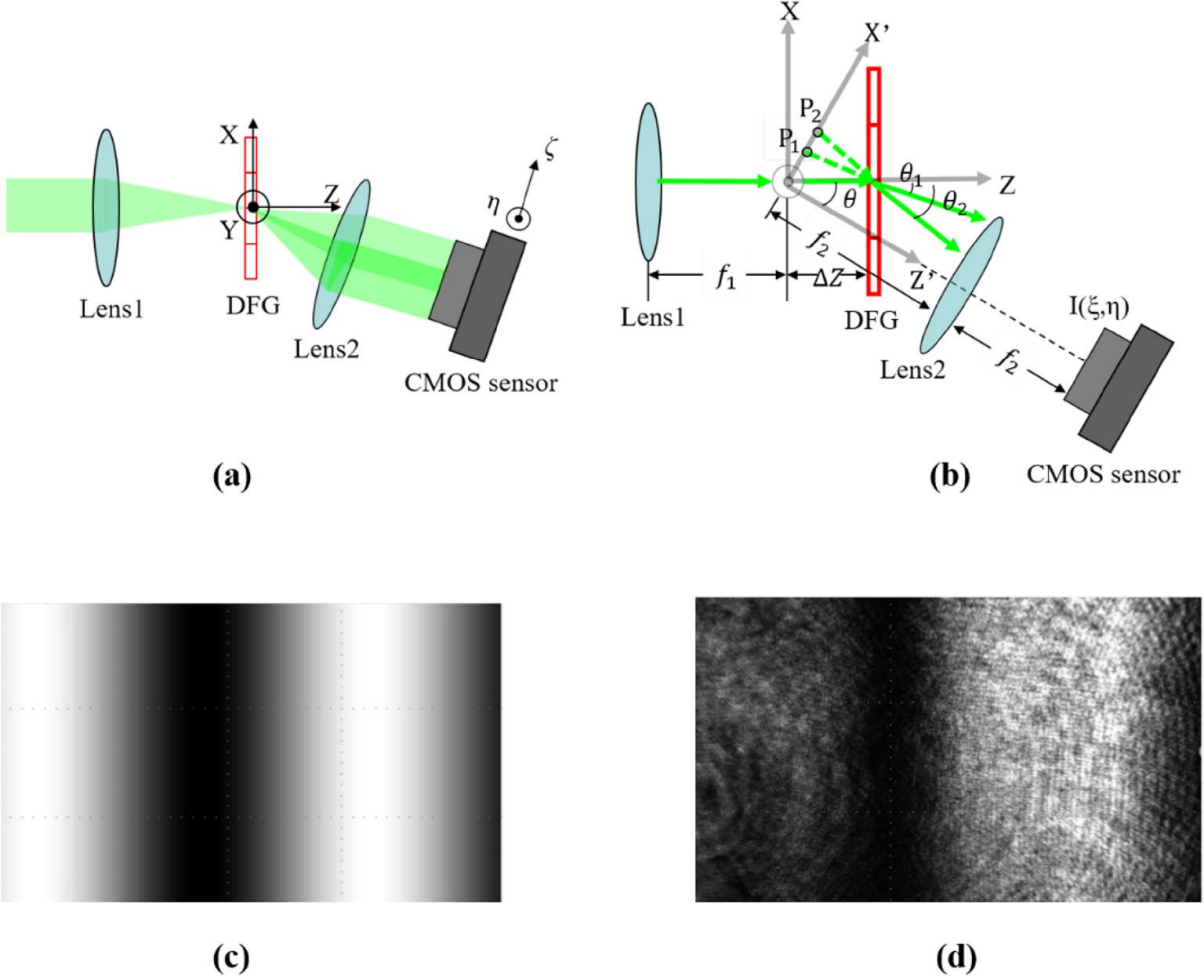
**(a)** The light path for the DFGSI, **(b)** the geometry, **(c)** the simulation of the interferogram by the two diffracted lights on the CMOS image sensor, and **(d)** the corresponding experimental observation.

DFGSI is a shearing interferometer that uses a DFG, as shown in Fig. 1a. The grating records two gratings with different spatial frequencies, one of which differs slightly from the other. The different spatial frequencies cause different diffraction angles so that the two first-order diffracted waves laterally sheared from each other and form shearing interferometry. The diffracted waves are sent to their Fourier transform planes on a CMOS image sensor. As illustrated in Fig. 1b, we derive the irradiance (I) on the CMOS image sensor^53,57^

1 $$I\left(\xi ,\eta \right)={\left|\begin{array}{c}\mathrm{exp}[i\Delta {\phi }_{0}+\frac{i\pi \Delta z\mathrm{cos}\left(\theta -{\theta }_{1}\right)\left({\xi }^{2}+{\eta }^{2}\right)}{\lambda {f}_{2}^{2}}+\frac{i2\pi\Delta z\mathrm{sin}\left(\theta -{\theta }_{1}\right)\xi }{\lambda {f}_{2}}]U\left(\frac{{f}_{1}}{{f}_{2}}\left[\xi -{f}_{2}\mathrm{sin}({\theta }_{1}-\theta )\right],\frac{{f}_{1}\eta }{{f}_{2}}\right)\\ +\mathrm{exp}[i\Delta {\phi }_{x}+\frac{i\pi \Delta z\mathrm{cos}\left(\theta -{\theta }_{2}\right)\left({\xi }^{2}+{\eta }^{2}\right)}{\lambda {f}_{2}^{2}}+\frac{i2\pi\Delta z\mathrm{sin}\left(\theta -{\theta }_{2}\right)\xi }{\lambda {f}_{2}}]U\left(\frac{{f}_{1}}{{f}_{2}}\left[\xi -{f}_{2}\mathrm{sin}({\theta }_{2}-\theta )\right],\frac{{f}_{1}\eta }{{f}_{2}}\right)\end{array}\right|}^{2},$$

where Δz is the distance between the front Fourier plane and DFG, θ is the bending angle between the optical axis of the Lens 1 and the Lens 2, θ_1_ and θ_2_ are the first-order diffracted lights, ξ and η are the coordinates in the image-sensor plane, f_1_ and f_2_ are the focal lengths of lens 1 and lens 2, respectively; Δϕ_0_ is the initial phase difference between the two diffracted waves induced by the phase relation of the two gratings, Δϕ_x_ is the relative phase shift induced by the DFG displacement (Δx) along the x-axis, and can be expressed as,

2 $${\Delta \phi }_{x}=\frac{2\pi }{\lambda }\left(\mathrm{sin}{\theta }_{1}-\mathrm{sin}{\theta }_{2}\right)\Delta x,$$

where λ is the wavelength.

Equation (2) is the key point in the interferometer. The shifting amount along the x-axis is a way to perform a phase shift between the two diffracted waves. Figure 1c, d show the simulated interferogram and experimental observations with the corresponding shifting amount. The experimental result and the simulation results are almost the same, except some ring-trace noise that is mainly induced by the multi-reflection of the Lenses and the cover glass of the CMOS sensor. This shows DFG displacement (Δx) can effectively induce phase shifts (${\Delta \phi }_{x}$) between the two diffracted waves. This property is useful for optical testing to retrieve the tested wavefront and is useful for 2D data processing to retrieve multi-level phase signals. Applications of 2D data processing include holographic data storage and other systems that need to encode multi-level phases in a 2D signal.

## Characteristics

The characteristics of the DFGSI with built-in phase-shifting interferometry (DFGSI-PSI) can be summarized as follows: (1) the PSI is a built-in function in the algorithm that allows for precise phase retrieval for each location/pixel; (2) the retrieved phase signal is acquired through differentiation, and the true phase can be obtained after the post-integral of the phase along the shifting direction; (3) the differentiation is generated by phase subtraction by the neighboring pixels, allowing shearing interferometry to alleviate a slow-varying phase error across the whole field. As this type of DFGSI-PSI is a novel interferometer, we discuss its detailed characteristics as follows. The differential phase value $\mathrm{\varphi }\left(\xi ,\eta \right)$ between the neighbor pixels are calculated from the irradiance of the 4-steps interferogram in each pixel using the equation that is the same as the PSI

3 $$\left\{\begin{array}{c}\mathrm{\varphi }\left(\xi ,\eta \right)={\mathrm{tan}}^{-1}\left(\frac{{I}_{1.5\pi }\left(\xi ,\eta \right)-{I}_{0.5\pi }\left(\xi ,\eta \right)}{{I}_{0}\left(\xi ,\eta \right)-{I}_{\pi }\left(\xi ,\eta \right)}\right):0\le \mathrm{\varphi }\left(\xi ,\eta \right)<\pi , { I}_{1.5\pi }\left(\xi ,\eta \right)-{I}_{0.5\pi }\left(\xi ,\eta \right)\ge 0 \\ \mathrm{\varphi }\left(\xi ,\eta \right)={\mathrm{tan}}^{-1}\left(\frac{{I}_{1.5\pi }\left(\xi ,\eta \right)-{I}_{0.5\pi }\left(\xi ,\eta \right)}{{I}_{0}\left(\xi ,\eta \right)-{I}_{\pi }\left(\xi ,\eta \right)}\right):\mathrm{\varphi }\le\upphi \left(\xi ,\eta \right)<2\pi , { I}_{1.5\pi }\left(\xi ,\eta \right)-{I}_{0.5\pi }\left(\xi ,\eta \right)<0\end{array}\right.,$$

where ${I}_{0}\left(\xi ,\eta \right)$, ${I}_{0.5\pi }\left(\xi ,\eta \right)$, ${I}_{1.5\pi }\left(\xi ,\eta \right)$, and ${I}_{2\pi }\left(\xi ,\eta \right)$ are the irradiance distributions of the 4-steps interferogram when the phase shift ${\Delta \phi }_{x}$ induced by Δx are 0, 0.5π, π, and 2π.

**Figure 2:**
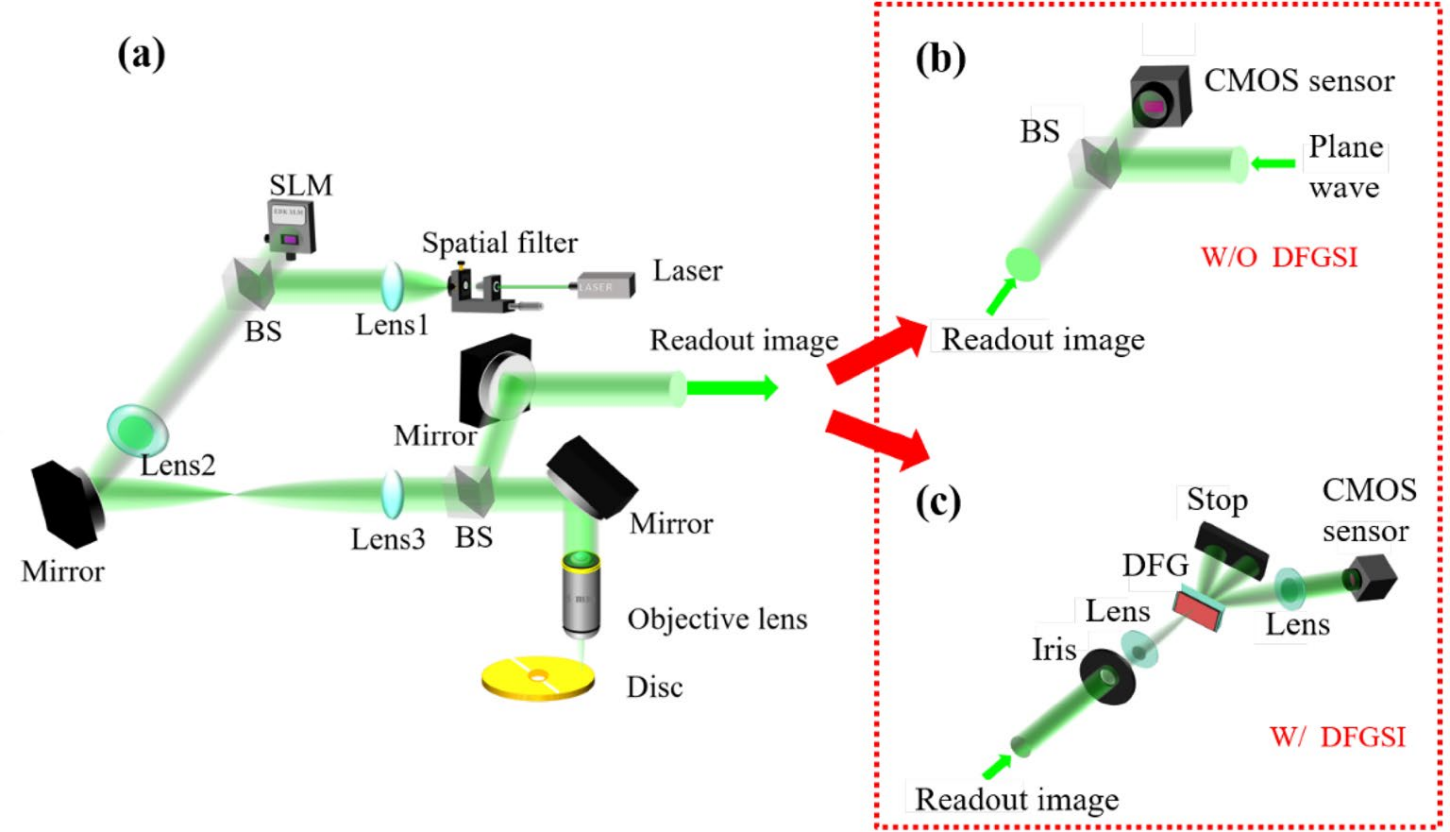
**(a)** The setup of a holographic data storage system, **(b)** a general PSI to retrieve the signal phase, and **(c)** the proposed DFGSI-PSI and spatial light modulator (SLM), which encodes the 2D multi-level phase.

To describe the characteristics of DFGSI-PSI for data processing, a volume holographic storage (VHS) system is introduced as an example, as shown in Fig. 2a. The readout signal can be sent to a general PSI or DFGSI-PSI to retrieve the signal phase (Fig. 2b, c). In contrast to the general PSI, DFGSI-PSI does not require an additional reference wave.

**Figure 3:**
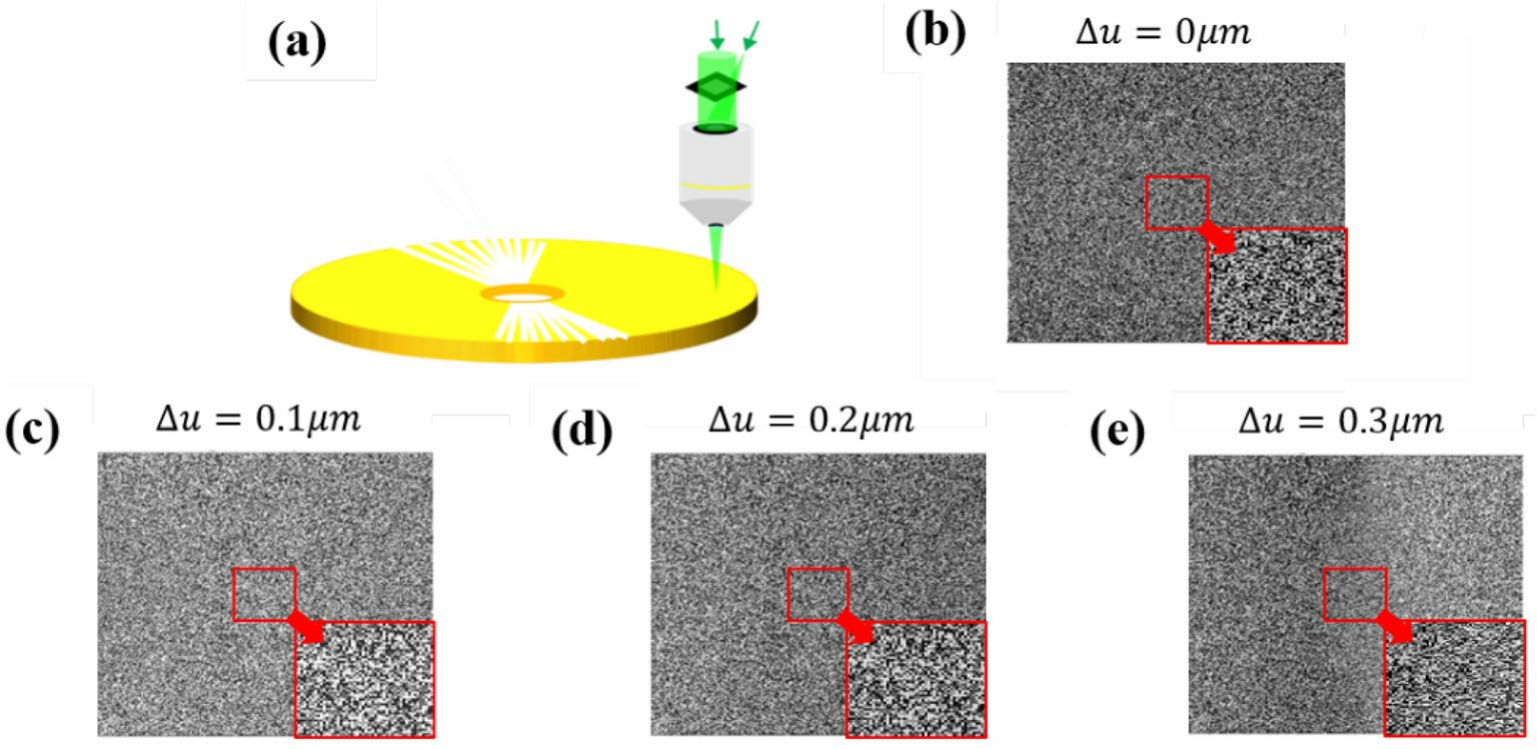
Simulations of the readout signal with phase encoding. **(a)** The schematic diagram of the pickup head, and the readout signal when the disc is displaced for **(b)** 0 μm, **(c)** 0.1 μm, **(d)** 0.2 μm, **(e)** 0.3 μm.

The VHS system is an off-axis recording system in which the 2D phase signal and spherical reference wave are sent to the recording disc through the objective lens. In the readout process, only the spherical reference wave is used to illuminate the disc at a precise incident angle and/or location, and the diffracted wave is reflected by the backside mirror of the disc and is sent for phase retrieval. Holographic multiplexing is applied at each recording location on the disc to increase the storage capacity, however different locations across the disc were utilized. Thus, during the readout process, the disc rotated for this purpose. Because of the Bragg condition, only a limited amount of rotation is permitted for effective diffraction. Therefore, a small amount of rotation corresponds to a small lateral displacement. The parameters in the simulation are as follows: the wavelength is 532 nm, the disc thickness is 2 mm, the focal length of the objective lens is 4 mm, the pixel size of the SLM is 6.4 μm, the pixel size of the CCD is 2.176 μm, the number of pixels number of the SLM are 1080 × 1080, and an effective pixel contains 4 × 4 SLM pixels. Figure 3 shows the simulations of the readout signals upon displacement of the disc (Δu). The displacement of the disc induces a slow-varying phase error across the signal plane^57^. The slow-varying phase error can be alleviated through the inherent characteristics of the shearing interferometry. This is because shearing interferometry is used for differentiation between adjacent pixels. Thus, the interferogram shows only the slope of the phase error, rather than the phase error itself. The phase differentiation property requires a post integral along the shearing direction to obtain the original signal. If phase errors occur in the interferogram, the integral will accumulate the phase errors. To overcome this problem, we propose the use of a pre-integral (PI) process to pre-process the input signal. For the original phase-only signal ${\varphi }_{s}\left({i}_{0},j\right)$, we apply a pre-signal process

4 $${\varphi }_{PI}\left(i,j\right)=\sum_{{i}_{0}=1}^{{i}_{0}=i-1}{\varphi }_{s}\left({i}_{0},j\right),$$

**Figure 4:**
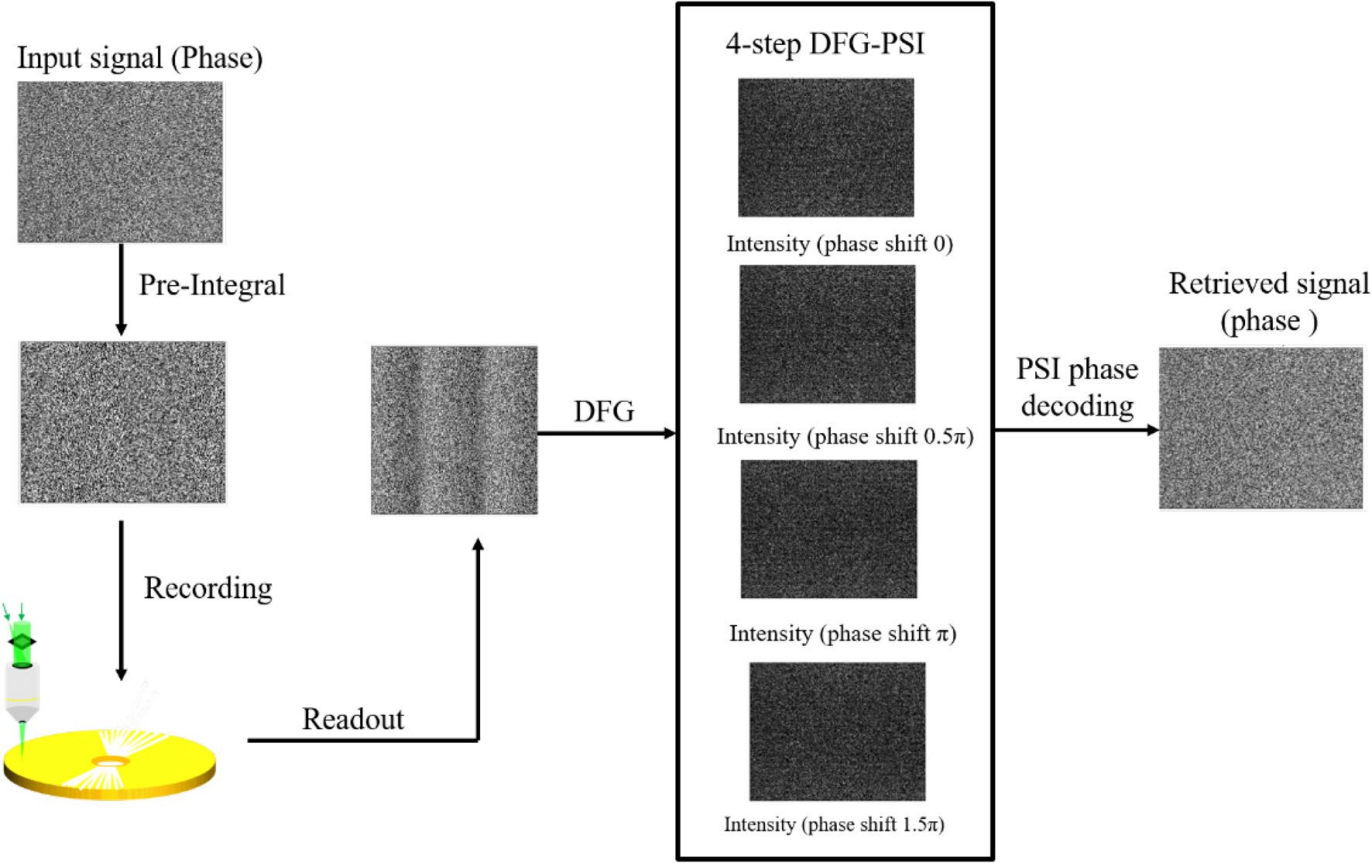
The flowchart of the proposed pre-integral-DFGSI-PSI.

Thus, the proposed interferometer is called pre-integral-DFGSI-PSI, which means that the pre-integral is applied to DFGSI-PSI to skip the post-integral of the decoded phase. Pre-integral-DFGSI-PSI cannot be applied in optical testing for an unpredicted wavefront, but it can be effectively used in data storage, communication, or other parallel image processing to increase the capability to retrieve multi-level encoded phases for a 2D phase signal. A flow chart of the complete interferometer for phase retrieval using a VHS system is shown in Fig. 4.

## Simulation and experiment

**Figure 5:**
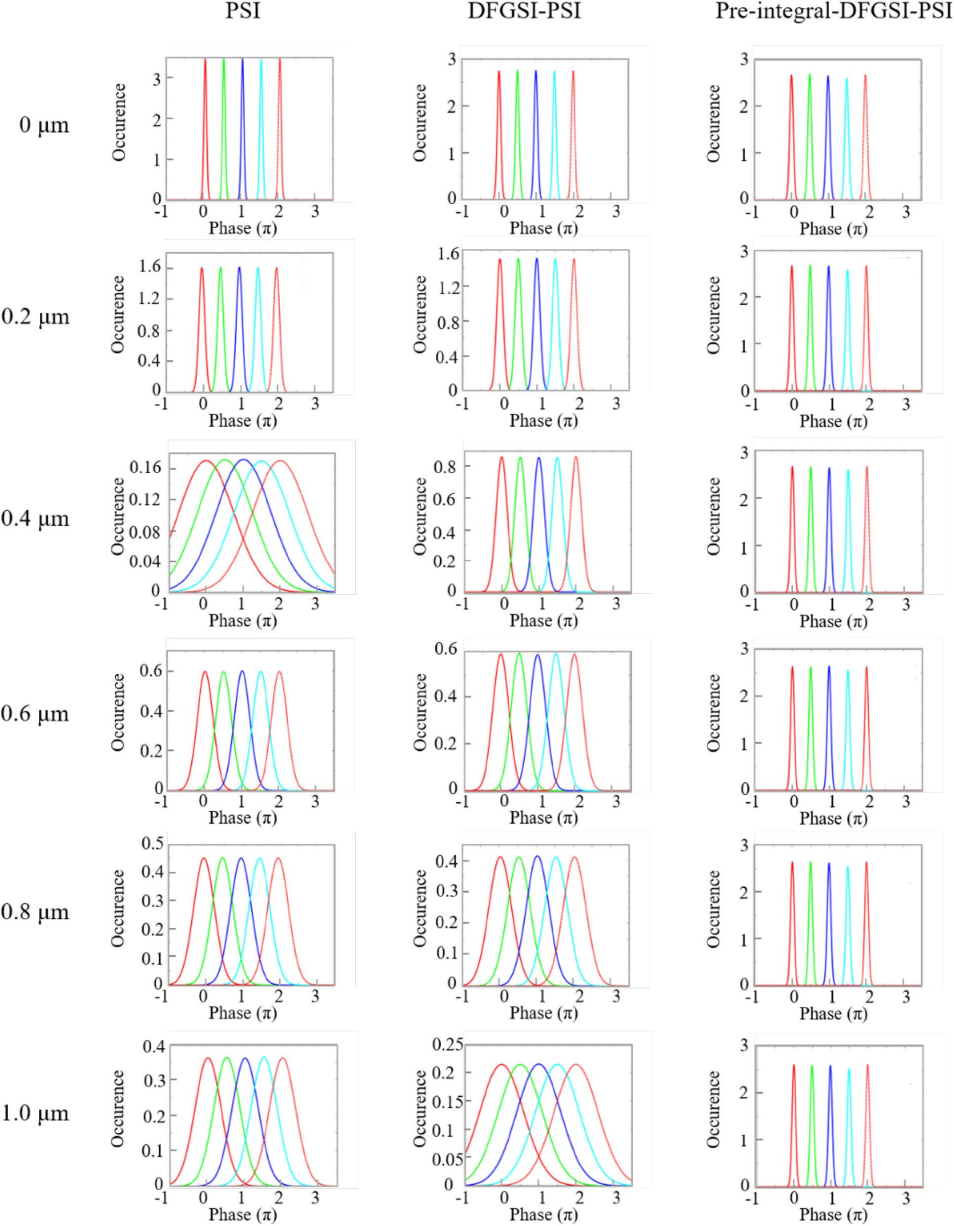
Simulation for the decoded phase signals of the PSI, DFGSI-PSI, and pre-integral-DFGSI-PSI under different disc rotation amounts of 0 μm, 0.2 μm, 0.4 μm, 0.6 μm, 0.8 μm, and 1.0 μm. Where the horizontal axis of each picture is retrieved phase, the vertical axis of each picture is the count of occurrence, the bright-red curve is the histogram for the coding signal 0, the green curve is the histogram for the coding signal 0.5π, the deep-blue curve is the histogram for the coding signal π, the light-blue curve is the histogram for the coding signal 1.5π, and the light-red curve is the histogram for the coding signal 2π.

**Figure 6:**
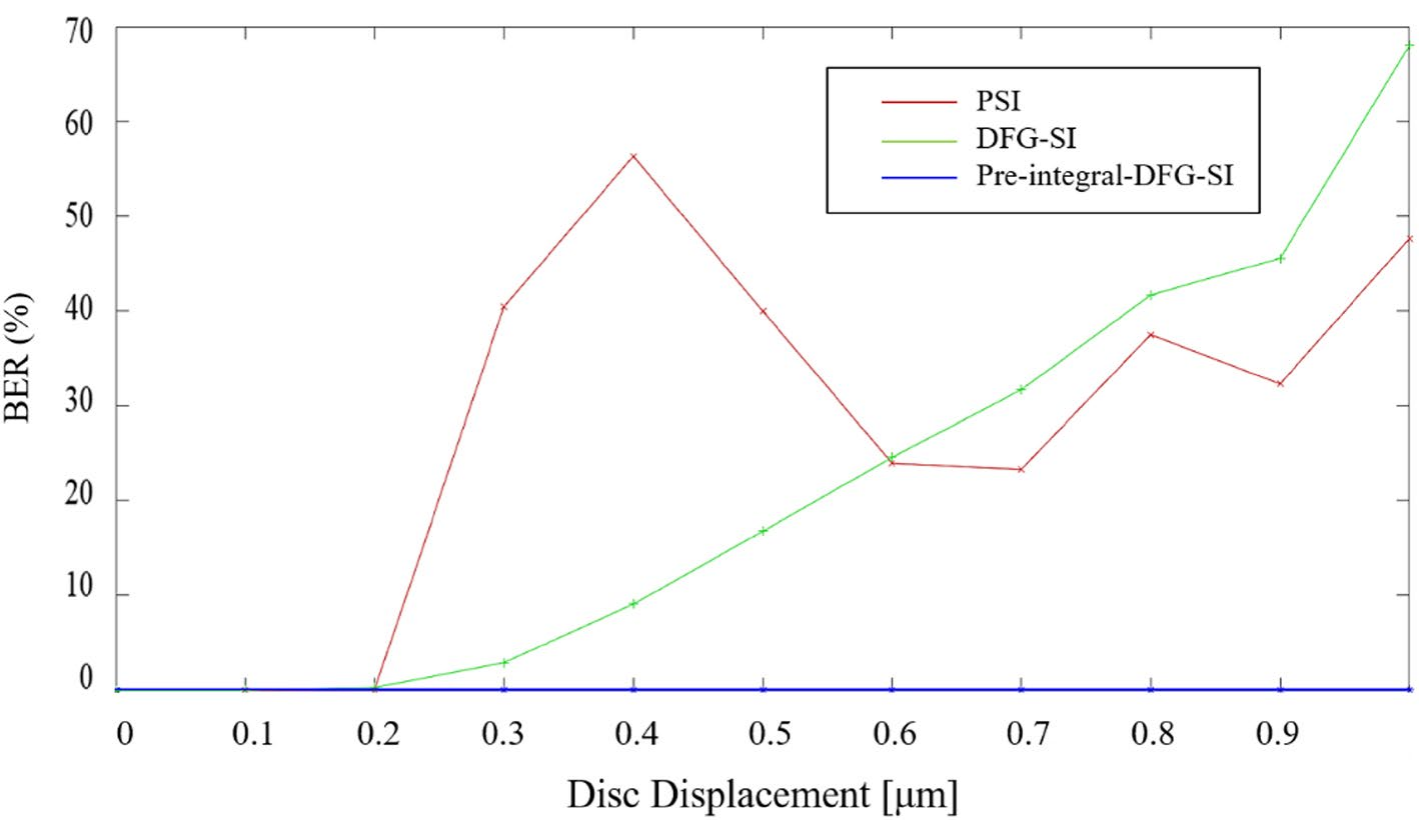
The calculated BER’s for the three types of the interferometer.

The simulation is based on the same condition as stated in the previous section, and it is used to examine whether the proposed pre-integral-DFGSI-PSI is a superior interferometer in 2D phase retrieval^57^. In the following, PSI means that a general 4-step phase-shifting algorithm is applied. DFGSI-PSI means that the DFG algorithm is used for phase shifting, but a post-phase integral is required to retrieve the original phase signal. Pre-integral-DFGSI-PSI means that an additional pre-integral process is applied before the signal is sent to the record. Thus, the phase signal can be retrieved after phase decoding using phase-shifting interferometry. To examine the resistance to the induced phase error in the readout process, the retrieved phase signal is subjected to different readout conditions with different disc rotation amounts ranging from 0 to 1.0 μm, as shown in Fig. 5. Figure 6 shows the calculation of the bit error rate (BER) for the three cases. In Figs. 5, 6, we can see that the general PSI has less resistance to the phase error. DFGSI-PSI, owing to the characteristic of shearing interferometry, has a higher resistance to phase errors. However, the post-phase-integral accumulates the phase error once a phase error occurs. From the high-contrast retrieved phases in Fig. 6, pre-integral-DFGSI-PSI exhibits a high resistance in phase error even when the disc rotates for a lateral displacement of 1.0 μm.

**Figure 7:**
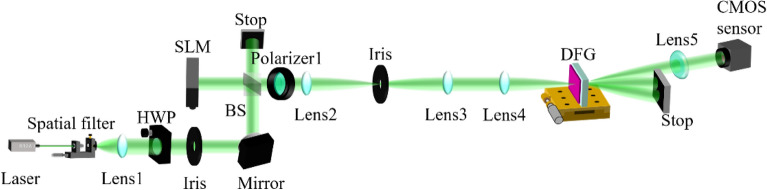
The experimental setup. BS, beam splitter; HWP, half-wave plate.

**Figure 8:**
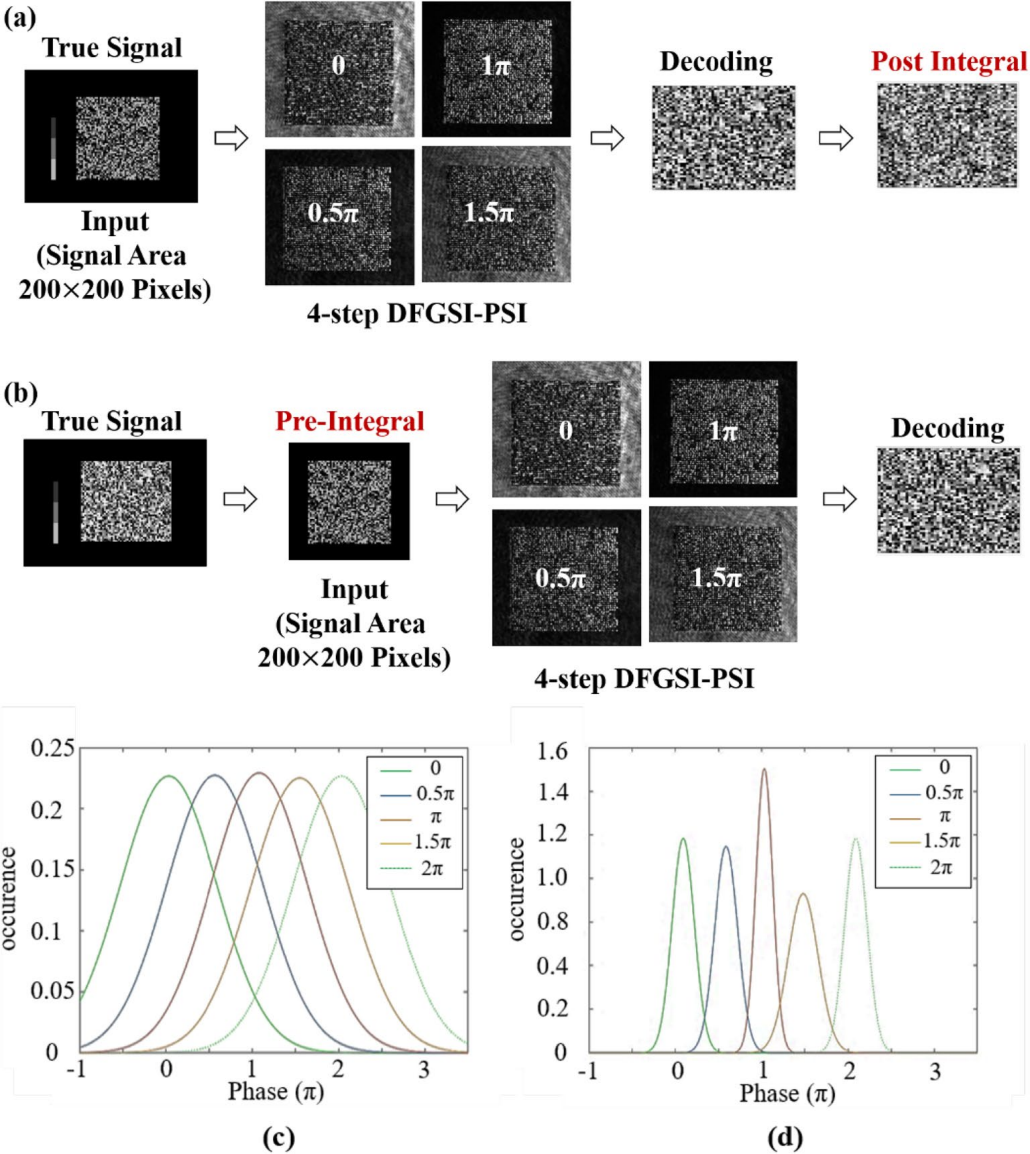
**(a)** The experiment of DFGSI-PSI with post integral, **(b)** the experiment of pre-integral–DFGSI-PSI, (**c**) the experimental results of phase retrieval of DFGSI-PSI with post integral, and **(d)** the experimental results of phase retrieval of pre-integral—DFGSI-PSI.

A similar experiment to prove the capabilities of pre-integral-DFGSI-PSI in 2D data processing was conducted using an experimental setup shown in Fig. 7, where the wavelength of the laser was 532 nm, the focal lengths were 105 mm (Lens1), 300 mm (Lens2), (300 mm (Lens3), 50 mm (Lens4), and 85 mm (Lens5); the angular deviation of the first-order diffraction of the two gratings was 0.0287°. A phase-type SLM by Jasper Display, JD955B, was used to encode the 2D phase on the incident light. The phase-encoded light wave passed through a DFG, which was attached at a precise translation stage, and the diffracted light was incident on a CCD image sensor. DFG was used to perform shearing and phase-shifting interferometry. A comparison of the experimental procedures of DFGSI-PSI with the post-integral and pre-integral-DFGSI-PSI is shown in Fig. 8. As shown in Fig. 8a, if the system was without PI, the input signal was the true signal without any processing. Through 4-step DFGSI-PSI, the signal phase was retrieved through a four-step DFGSI-PSI. Because the phase was the result of differentiation, an additional phase integral was required to recover the initial signal. The post integral can accumulate phase errors, so that the errors is widely spread. As shown in Fig. 8b, if the system was with PI, the input signal was through the pre-integral of the original signal. The true signal phase was retrieved using the same four-step DFGSI-PSI. The phase error is alleviated because it did not require a post integral, resulted in a sharp decoded signal phase, as shown in Fig. 8c, d, where the bit error rate (BER) of DFGSI-PSI with post integral was 0.6600, and that of pre-integral-DFGSI-PSI was 0.0626. This demonstrates the capability of the proposed pre-integral-DFGSI-PSI methods.

## Conclusions

In this paper, we first discuss DFGSI, which is a special interferometer with an appropriate design in a double-frequency grating to perform shearing interferometry. We then derive a built-in function of phase-shifting interferometry in DFGSI, where phase-shifting interferometry can be performed by laterally displacing the DFG. Therefore, the novel interferometer is referred to as the DFGSI-PSI. The advantages include two important functions that can be integrated into a device. The first function is the robust of phase retrieval, and it has the two benefits: (1) It doesn’t require an additional reference wave to retrieve the phase, and is robust in dynamic circumstances; (2) It can remove slow-varying phase errors following interferometer infection. The second function is a built-in function of phase shifting, which has been identified as one of the best techniques to accurately retrieve the multi-level phase. This type of new interferometer will be helpful in optical testing in dynamic circumstances with a common path and without an additional reference wave.

In addition to serving as an interferometer for optical testing, DFGSI-PSI is useful for retrieving the stored/carried phases in 2D data storage or 2D free-space communication. To increase the resistance to phase errors that could accumulate in the shearing interferometry, we propose a pre-integral process. In simulations and experiments, the pre-integral-DFGSI-PSI is proved to be more robust with a lower BER.

In summary, DFGSI-PSI is a powerful approach in optical testing, and pre-integral-DFGSI-PSI is an effective approach for retrieving multi-level phase signals, which increases the robustness of data in 2D optical storage and communication.

## References

[1] Heanue JF, Bashaw MC, Hesselink L (1994). Volume holographic storage and retrieval of digital data. Science.

[2] Coufal, H. J., Psaltis, D. & Sincerbox, G. T. eds. *Holographic Data Storage* (Springer, 2000).

[3] Wang J (2016). Investigation of the extraordinary null reconstruction phenomenon in polarization volume hologram. Opt. Exp..

[4] Nobukawa T, Nomura T (2016). Multilevel recording of complex amplitude data pages in a holographic data storage system using digital holography. Opt. Exp..

[5] Lin X (2020). Frequency expanded non-interferometric phase retrieval for holographic data storage. Opt. Exp..

[6] Takabayashi M, Okamoto A, Tomita A, Bunsen M (2011). Symbol error characteristics of hybrid-modulated holographic data storage by intensity and multi-phase modulation. Jpn. J. Appl. Phys..

[7] Kim KT, Cho BC, Kim ES, Gil SK (2000). Performance analysis of phase-code multiplexed holographic memory. Appl. Opt..

[8] Yu YW, Shu CM, Sun CC, Hsieh PK, Yang TH (2019). Optical servo with high design freedom using spherical-wave Bragg degeneracy in a volume holographic optical element. Opt. Exp..

[9] Lin SH (2014). Volume polarization holographic recording in thick photopolymer for optical memory. Opt. Exp..

[10] Wang M (2017). LCoS SLM study and its application in wavelength selective switch. Photonics.

[11] Wang J (2016). Advances in communications using optical vortices. Photon. Res..

[12] Fried DL (1967). Optical heterodyne detection of an atmospherically distorted signal wave front. Proc. IEEE.

[13] Ly-Gagnon D-S, Tsukamoto S, Katoh K, Kikuchi K (2006). Coherent detection of optical quadrature phase-shift keying signals with carrier phase estimation. J. Lightw. Technol..

[14] Zhang, C., Uyama, K., Zhang, Z., Jin, L. & S. Y. Set, and S. Yamashita, *Recent trends in coherent free-space optical communications*. In *Proceedings of the SPIE* **11712**, 117120M (2021).

[15] Borcea L, Garnier J, Sølna K (2020). Multimode communication through the turbulent atmosphere. J. Opt. Soc. Am. A Opt. Image Sci. Vis..

[16] Gibson G (2004). Free-space information transfer using light beams carrying orbital angular momentum. Opt. Exp..

[17] Wang J (2012). Terabit free-space data transmission employing orbital angular momentum multiplexing. Nat. Photon..

[18] Yao AM, Padgett MJ (2011). Orbital angular momentum: origins, behavior and applications. Adv. Opt. Photon..

[19] Willner AE (2015). Optical communications using orbital angular momentum beams. Adv. Opt. Photon..

[20] Kaushal H, Kaddoum G (2017). Optical communication in space: challenges and mitigation techniques. IEEE Commun. Surv. Tutor..

[21] Smutny, B. *et al.* In-orbit verification of optical inter-satellite communication links based on homodyne BPSK. *Proc. SPIE* **6877** (2008).

[22] Zhang S, Kam P-Y, Changyuan Yu, Chen J (2010). Decision-aided carrier phase estimation for coherent optical communications. J. Lightw. Technol..

[23] Koenig S (2013). Wireless sub-THz communication system with high data rate. Nat. Photon..

[24] Colavolpe G, Foggi T, Forestieri E, Prati G (2009). Robust multilevel coherent optical systems with linear processing at the receiver. J. Lightw. Technol..

[25] Toselli I, Gladysz S (2020). Improving system performance by using adaptive optics and aperture averaging for laser communications in oceanic turbulence. Opt. Exp..

[26] Chen M, Liu C, Rui D, Xian H (2018). Performance verification of adaptive optics for satellite-to-ground coherent optical communications at large zenith angle. Opt. Exp..

[27] Wang Y (2018). Performance analysis of an adaptive optics system for free-space optics communication through atmospheric turbulence. Sci. Rep..

[28] Carrizo CE, Calvo RM, Belmonte A (2018). Intensity-based adaptive optics with sequential optimization for laser communications. Opt. Exp..

[29] Zhang, S. *et al.* Extending the detection and correction abilities of an adaptive optics system for free-space optical communication. *Opt. Commun.* **482** (2021).

[30] Daoman, R. *et al.* Application of adaptive optics on the satellite laser communication ground station. *Opt. Electron. Eng.* **45** (2018).

[31] Woerdemann M, Alpmann C, Esseling M, Denz C (2013). Advanced optical trapping by complex beam shaping. Laser Photon. Rev..

[32] Greivenkamp, J. E. & Bruning, J. H., Chap. 14. Phase shifting interferometry. In *Optical Shop Testing*, 2nd ed. (ed. Malacara, D.) (Wiley, New York, 1992).

[33] Xu XF (2007). Blind phase shift extraction and wavefront retrieval by two-frame phase-shifting interferometry with an unknown phase shift. Opt. Commun..

[34] Gil SK (2012). 2-step quadrature phase-shifting digital holographic optical encryption using orthogonal polarization and error analysis. J. Opt. Soc. Korea.

[35] de Groot P (1995). Derivation of algorithms for phase-shifting interferometry using the concept of a data-sampling window. Appl. Opt..

[36] Larkin KG, Oreb BF (1992). Design and assessment of symmetrical phase-shifting algorithms. J. Opt. Soc. Am. A.

[37] Deck LL, de Groot PJ (1998). Punctuated quadrature phase shifting interferometry. Opt. Lett..

[38] Freischlad K (1996). Large flat panel profiler. Proc. SPIE.

[39] Nobukawa T, Katano Y, Muroi T, Kinoshita N, Ishii N (2021). Reduction of spatio-temporal phase fluctuation in a spatial light modulator using linear phase superimposition. OSA Cont..

[40] Ronchi V (1964). Forty years of history of a grating interferometer. Appl. Opt..

[41] Wyant JC (1974). White light extended source shearing interferometer. Appl. Opt..

[42] Koliopoulos CL (1980). Radial grating lateral shear heterodyne interferometer. Appl. Opt..

[43] Strojnik, M., Paez, G. & Mantravadi, M., Chap. 4. *Lateral Shear Interferometers* Optical Shop Testing, 3rd edn. (ed. Malacara, D.) (Wiley, New Jersey, 2007).

[44] Falldorf C, Heimbach Y, von Kopylow C, Jüptner W (2007). Efficient reconstruction of spatially limited phase distributions from their sheared representation. Appl. Opt..

[45] Liang PY, Ding JP, Jin Z, Guo CS, Wang HT (2006). Two-dimensional wave-front reconstruction from lateral shearing interferograms. Opt. Exp..

[46] García-Torales G (2006). Experimental intensity patterns obtained from a 2D shearing interferometer with adaptable sensitivity. Opt. Commun..

[47] Casillas FJ, Davila A, Rothberg SJ, Garnica G (2004). Small amplitude estimation of mechanical vibrations using electronic speckle shearing pattern interferometry. Opt. Eng..

[48] Nomura T, Okuda S, Kamiya K, Tashiro H, Yoshikawa K (2002). Improved Saunders method for the analysis of lateral shearing interferograms. Appl. Opt..

[49] Ferraro P, De NS, Finizio A, Pierattini G (2002). Reflective grating interferometer: a folded reversal and shearing wave-front interferometer. Appl. Opt..

[50] Villa J, García G, Gómez G (2001). Wavefront recovery in shearing interferometry with variable magnitude and direction shear. Opt. Commun..

[51] De Nicola S, Ferraro P, Finizio A, Pierattini G (2001). Two-beam interferometer for measuring aberrations of optical components with axial symmetry. Appl. Opt..

[52] Chen F (2001). Digital shearography: state of the art and some applications. J. Electron. Imag..

[53] Yu YW, Xiao S, Cheng CY, Sun CC (2016). One-shot and aberration-tolerable homodyne detection for holographic storage readout through double-frequency grating-based lateral shearing interferometry. Opt. Exp..

[54] Bjorkholm JE, MacDowell AA, Wood OR, LaFontaine B, Tennant DM (1995). Phase-measuring interferometry using extreme ultraviolet radiation. J. Vac. Sci. Technol. B.

[55] Mercer CR, Creath K (1994). Liquid-crystal point-diffraction interferometer. Opt. Lett..

[56] Griffin DW (2001). Phase-shifting shearing interferometer. Opt. Lett..

[57] Yu YW (2020). Reduction of phase error on phase-only volume-holographic disc rotation with pre-processing by phase integral. Opt. Exp..

